# Whole-body DWI in practice

**DOI:** 10.1186/1470-7330-14-S1-O47

**Published:** 2014-10-09

**Authors:** Anwar Padhani, Dow-Mu Koh

**Affiliations:** 1 Paul Strickland Scanner Centre, Mount Vernon Hospital, Rickmansworth Road, Northwood, HA6 2RN, UK; 2 Dept. of Diagnostic Radiology, The Royal Marsden NHS Foundation Trust, Downs Road, Sutton, Surrey, SM2 5PT, UK

## 

Whole-body diffusion-weighted imaging (WB-DWI) can be used to assess malignancy throughout the body for tumour staging and is being applied for the assessment of treatment response. One of the current unmet needs in oncology is the assessment of metastatic bone disease and diffuse bone marrow involvement, for which WB-DWI appears highly promising. For WB-DWI to be deployed successfully, meticulous data acquisition is necessary to obtain the highest quality images possible. Image post-processing is required to display images in a manner that is easy for referring clinicians to understand, especially on serial follow-up studies. Image interpretation requires knowledge on what is being displayed on WB-DWI. Key to the establishment of WB-DWI in clinical practice is the recognition of interpretative pitfalls, which will be discussed in detail including false-positive and false-negative cases. The potential clinical applications for WB-DWI are illustrated with case examples.

